# A Comprehensive Hybrid Approach for Indoor Scene Recognition Combining CNNs and Text-Based Features

**DOI:** 10.3390/s25175350

**Published:** 2025-08-29

**Authors:** Taner Uckan, Cengiz Aslan, Cengiz Hark

**Affiliations:** 1 Department of Computer Engineering, Faculty of Engineering, Van Yuzuncu Yıl University, Van 65080, Turkey; 2 Department of Artificial Intelligence and Robotics, Van Yuzuncu Yıl University, Van 65080, Turkey; cengizaslanvanyyu@gmail.com; 3 Department of Computer Engineering, Faculty of Engineering, Inonu University, Malatya 44050, Turkey; cengiz.hark@inonu.edu.tr

**Keywords:** indoor scene recognition, EfficientNet, object recognition, deep learning, text classification

## Abstract

**Highlights:**

**What are the main findings?**

- Proposed an innovative two-channel hybrid model by integrating convolutional neural networks (CNNs) with a text-based classifier.
- Leveraged an extended dataset derived from multiple object recognition models, increasing input data diversity and achieving a text-based classifier accuracy of 73.30%. Achieved a significant improvement of 8.33% in accuracy compared to CNN-only models, with the hybrid model attaining an accuracy of 90.46%.

**What is the implication of the main finding?**

- Efficient and Scalable Methodology: Utilized EfficientNet for CNN-based feature extraction and Bag-of-Words for text representation, ensuring computational efficiency and scalability.
- Application Potential: Addressed challenges in indoor scene recognition, such as complex backgrounds and object diversity, demonstrating significant potential for applications in robotics, intelligent surveillance, and assistive systems.

**Abstract:**

Indoor scene recognition is a computer vision task that identifies various indoor environments, such as offices, libraries, kitchens, and restaurants. This research area is particularly significant for applications in robotics, security, and assistance for individuals with disabilities, as it enables the categorization of spaces and the provision of contextual information. Convolutional Neural Networks (CNNs) are commonly employed in this field. While CNNs perform well in outdoor scene recognition by focusing on global features such as mountains and skies, they often struggle with indoor scenes, where local features like furniture and objects are more critical. In this study, the “MIT 67 Indoor Scene” dataset is used to extract and combine features from both a CNN and a text-based model utilizing object recognition outputs, resulting in a two-channel hybrid model. The experimental results demonstrate that this hybrid approach, which integrates natural language processing and image processing techniques, improves the test accuracy of the image processing model by 8.3%, achieving a notable success rate. Furthermore, this study offers contributions to new application areas in remote sensing, particularly in indoor scene understanding and indoor mapping.

## 1. Introduction

Scene recognition aims to classify environments based on human-defined labels, providing essential spatial context for intelligent systems such as robotics, autonomous driving, and video surveillance [1]. It is generally divided into two main categories: indoor scene recognition and outdoor scene recognition. Outdoor scenes typically consist of similar objects such as streets, avenues, gardens, and homogeneous backgrounds. In contrast, indoor scenes contain a wide variety of objects at different angles and scales, including living rooms, bedrooms, restaurants, and heterogeneous backgrounds. This diversity makes indoor scene classification more challenging than outdoor scene recognition [2]. Indoor scene classification enhances the ability of service robots to navigate and communicate effectively within indoor environments. By accurately recognizing indoor scenes—such as offices, corridors, or meeting rooms—robots can make autonomous decisions about their actions [3]. Indoor scene recognition further enables robotic systems to reach designated targets and execute more complex tasks by leveraging contextual understanding of the environment. Therefore, it is essential for robots to identify the indoor area they are in and determine the most relevant target object. These robotic technologies benefit from the context-based semantic relationships between objects and their surrounding environments, thereby improving task performance [4]. In addition to robotics, the contextual information obtained from scene recognition can enhance decision-making in applications such as autonomous vehicles and intelligent video surveillance systems [1].

While many classification models achieve high accuracy in outdoor scene recognition, they often fail to deliver similar success in indoor scene recognition. Quattoni and Torralba attribute this limitation to the necessity of focusing not only on global features (e.g., corridors and room structures) but also on local features (e.g., objects and fine-grained details) within indoor environments [5]. Outdoor scenes typically include areas such as beaches, streets, and forests, which often share similar object patterns, whereas indoor scenes present a more complex visual structure with numerous objects of varying textures and scales, such as classrooms, living rooms, and bedrooms [2].

This study is motivated by the need to emphasize objects that convey critical contextual information in indoor scenes. It is observed that using a hybrid model—combining object-focused information with traditional visual features—can significantly improve recognition performance. Therefore, a two-channel hybrid model is proposed in which a text-based approach is integrated alongside convolutional neural networks to enhance performance in the indoor scene recognition task, which is traditionally addressed using CNN-based computer vision techniques.

### 1.1. Contribution and Novelty

- Hybrid Approach for Indoor Scene Recognition: An innovative two-channel hybrid model was proposed by integrating convolutional neural networks (CNNs) with a text-based classification pipeline to capture both visual and contextual semantics.
- Enhanced Data Diversity: The model leveraged an enriched input set derived from multiple object recognition algorithms, which expanded the vocabulary and contributed to achieving a text-based classifier accuracy of 73.30%.
- Improved Model Performance: The proposed hybrid framework outperformed the CNN-only baseline by 8.33%, reaching an overall accuracy of 90.46% on the MIT Indoor-67 dataset.
- Efficient and Scalable Design: EfficientNet was employed for deep visual feature extraction, while a Bag-of-Words representation was used for textual inputs, ensuring a balanced trade-off between computational efficiency and model scalability. A computationally efficient and implementation-friendly method was adopted using the CNN-based EfficientNet model for visual feature extraction and basic methods such as Bag-of-Words for text representation.
- Application Potential: By addressing core challenges in indoor scene recognition—including complex backgrounds and high object variability—the proposed hybrid model demonstrates strong applicability in domains such as service robotics, intelligent video surveillance, and context-aware assistive technologies.

### 1.2. Related Work

Early research on scene recognition primarily addressed the classification problem by leveraging low-level image features. These studies typically focused on basic scene classification tasks involving a limited number of classes, such as “indoor vs. outdoor” or “urban vs. rural,” and utilized low-level visual cues such as color histograms, edge detection, and texture descriptors. For instance, Vailaya et al. conducted a study that aimed to distinguish between cityscapes and natural landscapes using such low-level features, classifying scenes based on color distribution and edge information [6]. These initial studies established the foundation of the field by integrating basic visual features with conventional machine learning techniques. In subsequent work, the bag-of-visual-words approach gained prominence, providing a general representation of local features. Yang et al. extracted local features from images, transformed them into visual words, and then constructed histograms from these words to represent scenes [7]. Quattoni and Torralba later emphasized the importance of distinguishing between local and global features, especially for indoor scene classification. To address this, they developed prototype-based models that captured both feature types using candidate regions annotated with human-provided visual descriptions. Their model achieved improved recognition performance by combining local features—capable of differentiating objects—with global scene-level prototypes [5].

Before the advent of deep learning—particularly convolutional neural networks (CNNs)—scene recognition relied primarily on statistical machine learning techniques. CNNs have revolutionized scene recognition and many other computer vision tasks [8]. Zhou et al. emphasized that CNNs can effectively learn object-based relationships along with global features such as edges and colors, highlighting the role of intermediate representations in deep networks [9]. Chen et al. improved indoor scene recognition by constructing a word matrix based on object embedding techniques, which enhanced the top-five classification ranking from CNNs [10]. Miao et al. proposed the “Object to Scene” (OTS) approach, which leverages object features and their relationships for scene classification [11]. They enhanced accuracy by processing features extracted from an object segmentation network through an attention module. Nagarajan and Thanabal addressed the scene recognition task by categorizing object words—obtained from an object recognition algorithm—into “mandatory” and “desirable” groups, showing that this simple categorization can yield effective classification results [12]. Heikel and Espinosa-Leal approached scene recognition as a natural language processing problem, using the term frequency–inverse document frequency (TF-IDF) method to classify object words obtained from their trained recognition model [4]. Basu et al. developed a two-stage model that combines object recognition and CNNs by using features from the Mask-RCNN algorithm to classify five scene categories, achieving notable performance in indoor environments [13]. Khan and Othman introduced a dual-flow model for indoor classification: a global flow to extract contextual information at multiple scales using the VGG-16 architecture and a local flow to capture fine-grained scene details, resulting in a more comprehensive scene representation [3]. These studies underscore the necessity of incorporating object-level features in indoor scene recognition, highlighting their importance alongside global characteristics. Indoor scenes are inherently more complex than outdoor ones due to the diversity of objects in terms of size, shape, and spatial arrangement, making accurate classification more challenging [14]. The difficulty of indoor classification also arises from significant intra-class variation and, in some cases, subtle inter-class differences [15]. Sun et al. noted that this challenge stems from high intra-class diversity and low inter-class variability [16]. Consequently, rich contextual information becomes essential for reliable scene recognition, with a focus on objects proving vital in capturing this complexity [17].

The previously summarized studies in the literature highlight the necessity of focusing on local features—particularly objects—in addition to global or general features. These studies propose various methods to utilize local features in order to overcome the limitations of convolutional neural networks (CNNs). While CNNs are successful in capturing global spatial characteristics of images, they may struggle to provide the level of discrimination required for indoor scenes [3]. The primary motivation for incorporating text classifiers alongside CNNs in this study is to address this limitation by using object-based words that represent local features. The literature review reveals that no existing two-channel approach integrates both a text classifier and a CNN. Furthermore, unlike prior studies, as detailed in Section 4.2, this study combines words obtained from multiple object recognition models into a comprehensive corpus named the “Extended Model.” This corpus is then used to train a high-performing text classifier, which serves as a component of the proposed hybrid model. This study achieves a high level of accuracy in indoor scene recognition through this novel hybrid approach.

The dual-task multiscale model proposed by Jing Wen Li and colleagues in Scientific Reports achieved an overall accuracy (OA) of 98.4% by integrating squeeze-and-excitation (SE) attention and Transformer modules into the ResNet50 architecture outputs [18]. Similarly, the JCVT model developed by Chen Wang and his team—published on SSRN—employs a hybrid architecture combining ResNet50 and Vision Transformer (ViT), reporting a comparably high accuracy on the Indoor 67 dataset [19]. Table 1 provides a comparative overview of the features of the Transformer-based architectures and the proposed hybrid model.

## 2. Background

This section outlines the methods and technologies underlying the two-channel hybrid model, with further details provided in the subsequent section. The first channel utilizes features extracted from a convolutional neural network (EfficientNet), which approaches scene recognition as a computer vision task. The second channel leverages features from a text-based scene classifier developed using object recognition.

### 2.1. Convolutional Neural Networks—EfficientNet

As with many computer vision problems, Convolutional Neural Network (CNN)-based architectures are traditionally employed in indoor scene recognition tasks. EfficientNet, a CNN-based model, is recognized for its high accuracy and computational efficiency [20]. In this study, EfficientNet is preferred due to its scalability and the availability of multiple variants with different complexities and sizes. Unlike traditional models that typically scale only one dimension—depth, width, or resolution—EfficientNet introduces a compound scaling method that balances all three dimensions simultaneously (depth = *d*, width = *w*, resolution = *r*). This approach, formulated using a compound coefficient as shown below, enables the model to achieve higher accuracy with fewer parameters and reduced computational cost. The FLOPs (Floating Point Operations) value, which quantifies computational expense, is directly proportional to the depth, width, and resolution of the network, as illustrated in Equation (1) [21].

FLOPs ∝ d × w2 × r2(1)

d = αϕ, w = βϕ, r = γϕ(2)

The values “α, β, and γ” presented in Equation (2) represent the growth rates for the depth, width, and resolution of the model, respectively. The term “Φ” denotes a compound scaling factor that proportionally increases with model size, thereby affecting the computational load, speed, and accuracy. The constraints provided in Equation (3) are applied to determine appropriate values for these scaling parameters.

α⋅β2 ⋅ γ2 ≈ 2, α,β,γ > 0 (3)

**Figure 1:**
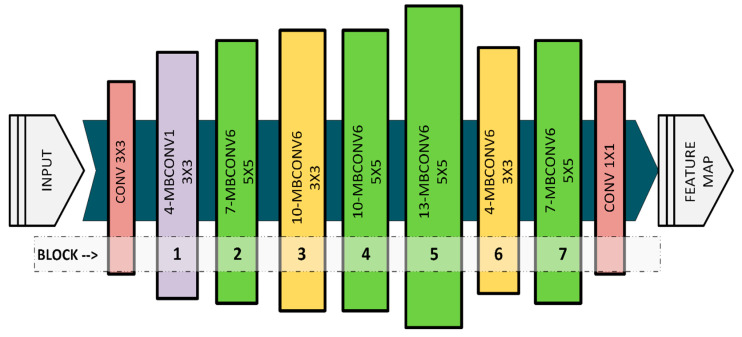
EfficientNet B7 model architecture.

With these constraints, increasing the width and resolution results in a quadratic rise in computational cost, while maintaining a balanced overall cost increase [21]. The operational structure of the EfficientNet-B7 architecture is illustrated in detail in Figure 1.

### 2.2. Text Classification

Text classification is a natural language processing task that aims to categorize text based on different aspects such as sentiment, type, topic, or scope [22]. Both traditional statistical methods and deep learning-based techniques can be employed for this purpose. Compared to traditional approaches, deep learning methods often yield more context-aware and relational results in text classification tasks [23]. Object recognition, on the other hand, is a computer vision task that seeks to identify objects within an image. Recent advancements in deep learning have significantly enhanced performance in this field. Moreover, object recognition serves as a foundational step for various downstream tasks, including object detection and tracking, image captioning, and semantic segmentation [24].

**Figure 2:**
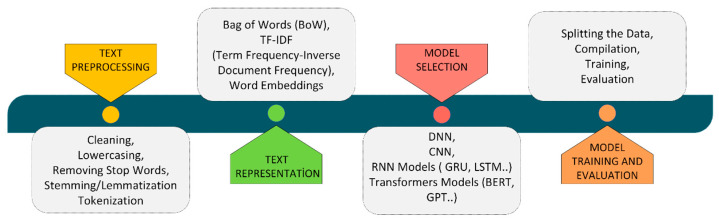
Text classification stages with deep learning.

In this study, object recognition algorithms are used to extract object names from scene images. These extracted names are then recorded as input data for the developed text-based model. The stages of the deep learning-based text classification process are summarized in Figure 2 below.

## 3. Proposed Approach

The proposed approach is based on the idea that objects within a scene can serve as distinctive features to describe that scene. A text-based scene classifier is constructed using object names extracted through object recognition algorithms. The features derived from this text-based classifier are then combined with those obtained from a convolutional neural network-based classifier to train a new hybrid model. It has been observed that this two-channel hybrid architecture, which integrates natural language processing and computer vision techniques, significantly improves performance.

### 3.1. CNN Based Scene Classification

**Figure 3:**
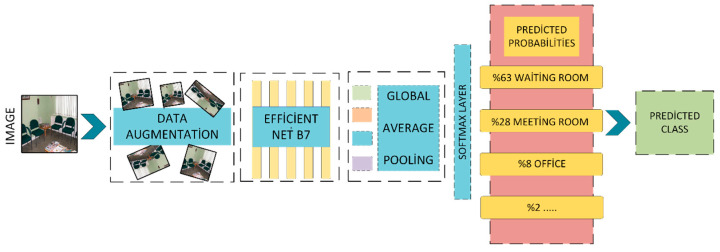
CNN-based scene classification architecture.

In this study, the EfficientNet model is chosen due to its effectiveness and efficiency in extracting visual features from indoor scenes. These visual features constitute the first channel of the proposed two-channel hybrid architecture. Scene images, augmented through data preprocessing techniques, are fed into the EfficientNet-based classifier, which has been pre-trained on the ImageNet dataset. The CNN-based scene classification architecture employed in this study is illustrated in Figure 3.

As shown in Table 2, the image input size is set to 600 × 600 pixels, in accordance with the EfficientNet-B7 model. Eighty percent of the data is allocated for training. The accuracy of the EfficientNet-based scene classifier (82.13%) is taken as the baseline in this study, and the goal is to improve this performance through the proposed hybrid model.

### 3.2. Text-Based Scene Classification

Text-based methods for scene recognition typically treat the objects present in a scene as words forming the input text. These object words are extracted using pre-trained object recognition models. In the literature, methodological differences mainly emerge at the vectorization stage when utilizing these words for scene classification. For instance, Heikel and Espinosa-Leal employed TF-IDF for vectorizing object words, whereas Chen and colleagues used word embedding techniques [4,10]. In this study, the preprocessed and tokenized text was vectorized using the bag-of-words method, which was chosen for its simplicity, interpretability, and effectiveness. To achieve a more balanced frequency distribution from the bag-of-words representation, 1 was added to each element before applying the natural logarithm. This transformation was intended to normalize the word frequency distribution, thereby mitigating the impact of highly frequent tokens and enhancing feature interpretability.

**Figure 4:**
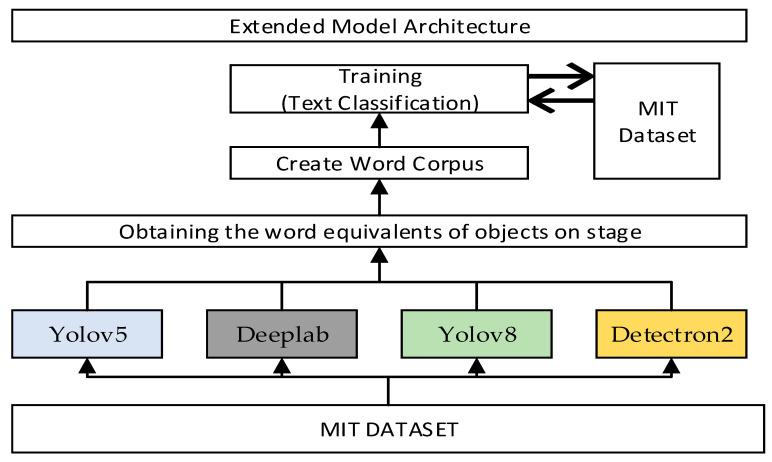
The architectural structure of the extended model.

For the text classifier, whose pseudocode is presented in Algorithm 1, the object recognition models detailed in Table 3 are utilized. The words extracted from these models are aggregated into a corpus to increase data volume and diversity and are used as input for model training—referred to as the “Extended Model” by the authors. The concept of the “Extended Model” is based on combining class labels obtained from different object recognition models (YOLOv5, YOLOv8, Detectron2, DeepLab) to create an enriched word sequence. Here, each model was run on the same scene images, and the object names detected in the scene were obtained independently. By combining the outputs of different models, a more comprehensive and diverse representation of objects is obtained for each scene. The expanded word sequence created as a result of this process forms the input for the text-based classifier. Therefore, the term “Expanded Model” does not refer to the combination of separate models trained on different datasets but rather the content-based integration of output information from multiple object recognition models operating on the same data. This concept aims to provide higher object diversity and contextual information in scene classification. The architecture of the extended model is shown in Figure 4.
**Algorithm 1.** Pseudocode of the text-based scene classifier.**Input**:  I : RGB image input of size (224 × 224 × 3)  T : Scene description or class-level text annotation **Output**:  ŷ : Predicted scene class label **Procedure**: **1. Visual Feature Extraction**:  CNN_feat ← CNN_Extractor(I)    # Example architecture: DenseNet121 (pretrained on ImageNet)    # Output dimension: ℝ^(2560) **2. Text Feature Extraction**:  Text_feat ← Vectorizer(T)    # Preprocess: Tokenize, stop-word removal, lowercase    # Text vector size = 15,000 (selected via max_features hyperparameter)    # Output dimension: ℝ^(15,000) **3. Normalization (optional but recommended for balanced fusion):**  CNN_feat ← Normalize(CNN_feat)     # L2 or Min-Max scaling  Text_feat ← Normalize(Text_feat) **4. Feature Fusion:**  Fused ← Concatenate(CNN_feat, Text_feat)    # Final shape: ℝ^(2560 + 15,000) = ℝ^(17,560)    # Concatenation performed along the feature axis **5. Fully Connected Classifier:**  h1 ← ReLU(FC1(Fused))     # FC1: ℝ^(17,560) → ℝ^(4096)  h2 ← ReLU(FC2(h1))      # FC2: ℝ^(4096) → ℝ^(1024)  logits ← FC3(h2)     # FC3: ℝ^(1024) → ℝ^(C), where C = number of classes  ŷ ← Softmax(logits) **Return ŷ**

The object words are cleaned of symbols and special characters, normalized, and then tokenized. The resulting word tokens are represented using the Bag-of-Words (BOW) method. To ensure a balanced distribution, 1 is added to each word frequency before applying the logarithmic transformation, as shown in Equation (4).

(4) $${Xfreq}^{\prime }=ln(1+Xfreq)$$

**Figure 5:**
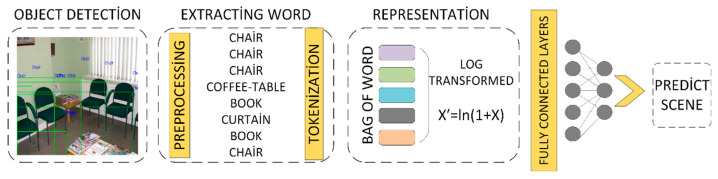
Text-based scene classification architecture.

This approach is preferred to prevent certain frequently repeated words in specific scenes (e.g., person in airport scenes, chair in auditorium or classroom scenes) from negatively affecting the performance of the text-based classifier [25]. The text-based classifier, referred to as the Extended Model, is trained using the prepared word representations and subsequently evaluated on the test data. The architecture of the Text-based scene classification is shown in Figure 5.

In deep learning, increasing the volume and diversity of input data enhances the generalization ability of trained models [16]. From this perspective, multiple object recognition models trained on different datasets were employed to expand both the quantity and variety of object words used as input for the text-based model. Unlike previous studies, this research constructed a comprehensive word corpus—referred to as the Extended Model—by leveraging multiple pre-trained object recognition models trained on diverse datasets. These models were selected from publicly available sources on the web to avoid the cost of retraining [26,27,28,29]. To ensure a broad object vocabulary, models trained on large-scale datasets with a wide range of classes were preferred.

The datasets on which the object recognition models were trained, along with the number of classes they contain, are presented in Table 3. Object recognition was performed on the “MIT Indoor Scene” dataset using the models listed in the table. The total number of detected objects, the average number of objects per image (calculated as the number of detected objects divided by the total number of images in the dataset, which is 15,620), and the total number of unique objects are also reported. The model referred to as the Extended Model was created by the authors by merging the object words extracted from all models into a single corpus.

When Table 3 is examined, it can be seen that Model-1, trained on the Object365 dataset, extracts an average of 20 objects per image, whereas Model-3, trained on the Open Images dataset, extracts fewer objects on average (16), despite having a larger number of object classes. This is likely because the Object365 dataset contains more classes that can be defined as indoor-related objects (e.g., various types of furniture). Based on this observation, it can be concluded that when developing a text-based scene recognition model using object recognition methods, the training datasets of object recognition algorithms should include classes that align with the characteristics of indoor scenes. Although Model-2 has fewer object classes, a lower average number of detected objects, and fewer unique objects compared to Model-1, it demonstrates higher performance. This may be attributed to the nature of the dataset on which Model-2 was trained. Specifically, the COCO-Stuff dataset includes both foreground objects (“things”) and background elements (“stuff”), which can provide a richer source of contextual information for indoor scene recognition. For instance, the Object365 dataset does not include the “wall” class, as it is considered part of the background texture rather than an object. In contrast, the COCO-Stuff dataset contains four distinct wall-related classes (wall-brick, wall-stone, wall-tile, wall-wood), offering more detailed background information. This additional context is believed to enhance the model’s ability by providing a more comprehensive understanding of the indoor environment.

**Figure 6:**
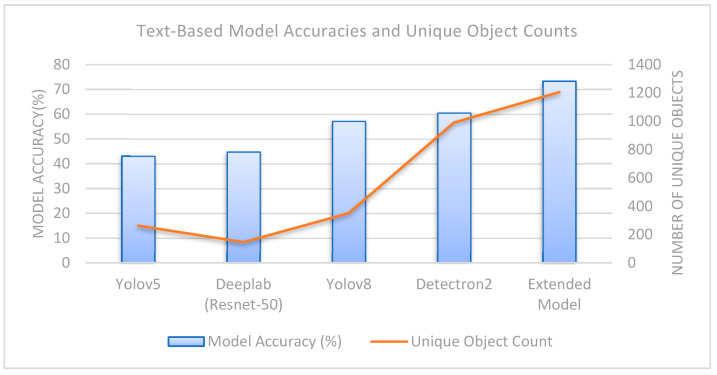
Comparison graph of text-based model accuracies and unique object counts.

In Figure 6, the success of the models trained with words of each object recognition model is compared. Since the extended model is superior to the other data in terms of input data, number of objects and object diversity (number of unique objects), it has the highest test success (73.30%) as expected. When the test success of the extended model tested on a challenging dataset such as MIT Indoor Scenes is considered, it can be said that the applied text-based method is successful on its own. The features extracted from this text classifier are used as the second channel in the proposed two-channel hybrid model.

Object labels in object recognition datasets often vary in naming conventions. Frequently, the same object is represented with alternative or descriptive terms. For example, in the LVIS dataset, the label for “person-human” includes alternatives such as “person,” “baby,” “child,” “boy,” “girl,” “man,” “woman,” and “human” under a single tag, whereas the Open Images dataset defines labels like “person,” “man,” “woman,” and “boy” separately. Since these object recognition algorithms are developed independently, managing label overlaps and distinctions among hundreds of terms becomes quite challenging. To simplify implementation and leverage the shared descriptive vocabulary, all alternative words are treated as distinct entries. This approach also helps in capturing commonly used relational words, which aligns with the hybrid model’s aim of extracting contextual information. For instance, while the tag “human” exists in the LVIS dataset, it is absent from the Open Images dataset. However, the latter includes 13 tags related to human body parts, such as “human arm,” “human beard,” “human body,” and “human ear.” Given such differences in naming conventions, where part–whole relationships vary, the most appropriate strategy is to tokenize compound terms like “human arm” into separate tokens, such as “human” and “arm,” thereby allowing the model to extract semantic meaning from each component. In summary, compound terms are decomposed into their individual components and treated as separate words to standardize inputs across different datasets with inconsistent labeling schemes. The experimental findings of the logarithmic transformation applied to word frequencies are presented in Table 4.

As seen in the table, the logarithmic operation aimed at normalizing the frequency distribution results in a significant increase in test accuracy.

### 3.3. Two-Channel Hybrid Model

**Figure 7:**
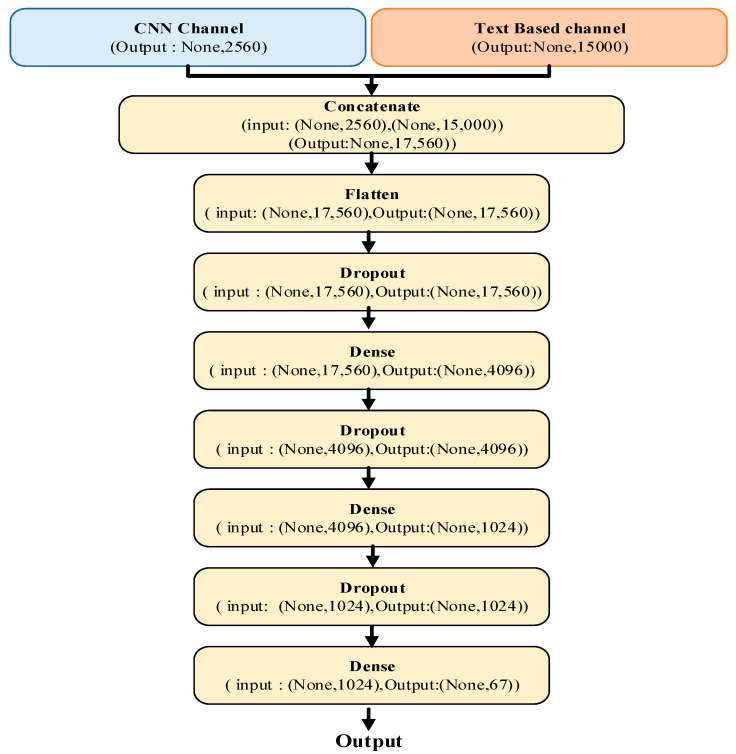
Layers of the proposed hybrid model.

The difficulties inherent in indoor scene recognition—such as complex backgrounds, a high number of objects, small intra-class differences, and large inter-class similarities—limit the performance of convolutional neural networks. Despite their success in identifying global spatial features, CNNs have limitations in capturing the fine-grained distinctions required for indoor scenes. To address this issue, a hybrid model was developed that focuses on local features, particularly objects. This hybrid model adopts a two-channel architecture, where features extracted from two different classifiers are combined. Feature extraction is performed using natural language processing techniques for the text classifier and computer vision techniques for the CNN-based classifier. These two feature sets are then merged via concatenation and trained together. Figure 7 illustrates the matrix transformations of the features from both channels through their respective layers.

**Figure 8:**
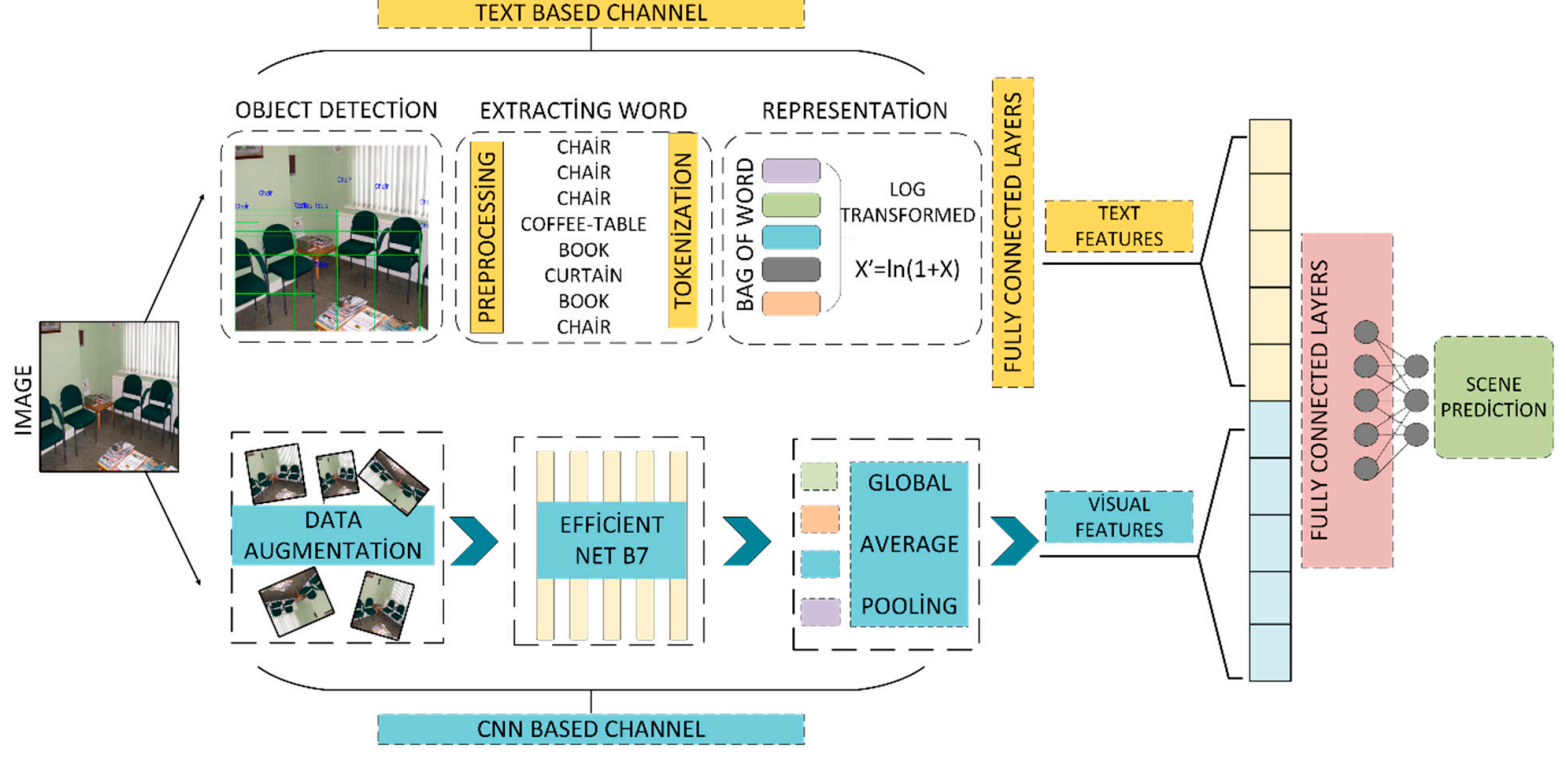
Processing stages of the proposed hybrid model.

The processing stages of the two-channel hybrid model are illustrated in detail in Figure 8. In the first channel, which is based on text-based feature extraction, the input consists of object names identified by object recognition algorithms from a given image. Before being used, these words are cleaned of punctuation and symbols and then subjected to standard text preprocessing steps, such as converting all characters to lowercase to ensure consistency. YOLO-based object recognition algorithms produce multidimensional tensors containing class labels, confidence scores, and location information (x, y, width, height) for objects detected in each image. These outputs are subjected to four basic preprocessing steps prior to analysis: (i) Object detections with confidence scores below a certain threshold are removed to filter out noise. (ii) The class label with the highest probability is selected for each object so that each detection is represented by a single category. (iii) The selected class labels were matched with the model’s dictionary and converted into text format to create textual data (e.g., chair, window, bottle). (iv) If there were multiple objects belonging to the same class, they were represented only once to make the texts unique. As a result of these steps, the object information obtained from the image was converted into a consistent and meaningful text input, and this structure was used in the text-based channel of the proposed hybrid model. This preprocessing step clarified object descriptions and enhanced the model’s contextual scene understanding. The tokenized words are represented using the Bag-of-Words (BoW) method, and their frequencies are transformed logarithmically to achieve a more balanced distribution. In the final stage, these word representations are passed through fully connected layers for classification. The features extracted from this text-based classifier are then utilized as one of the components of the hybrid model.

The second channel of the model processes visual features through a CNN-based architecture. Input images are first subjected to data augmentation, which is expected to enhance the model’s generalization capability. At this stage, EfficientNet-B7 is employed for visual classification. Additionally, a Global Average Pooling layer is integrated into the model to both emphasize the overall structure of the image and reduce the dimensionality of the feature map. The visual features obtained through this CNN-based pipeline constitute the second input channel of the hybrid model. In this study, only the basic concatenate approach was applied; alternative strategies such as attention, bilinear pooling, and gated fusion were excluded from the experimental scope. However, in future versions of the model, it is planned to systematically compare these methods and investigate normalizing transformations aimed at improving modality balance.

## 4. Experimental Studies

### 4.1. Dataset

**Figure 9:**
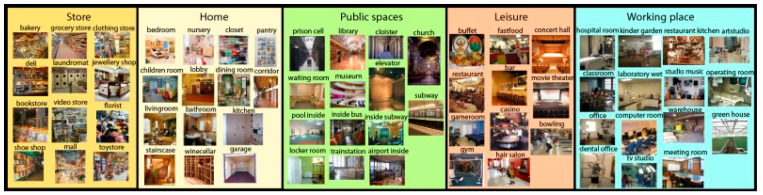
MIT Indoor Scenes dataset image [5].

As illustrated in Figure 9, the hybrid model developed in this study was trained and evaluated using the MIT Indoor Scene dataset. This dataset is known for its challenging nature, comprising 67 distinct classes that often exhibit similar contextual features—for instance, kitchen, restaurant kitchen, restaurant, and fast food restaurant. Due to these complexities, it has become a widely adopted benchmark in the literature for indoor scene recognition tasks and is considered a standard for assessing model performance. In this study, the dataset was split into 80% for training (12,496 images), 10% for validation (1562 images), and 10% for testing (1562 images) [5].

### 4.2. Evaluation Metrics

The accuracy calculations of the model are shown in Equation (5), which expresses the percentage based on the basic Accuracy (%) formula. “y” represents the true probability distribution, and “ŷ” represents the predicted probability. “C” represents the total number of elements, and “i” represents the element index [30]:

(5) $$\mathrm{Accuracy}=\frac{\sum_{i=1}^{C}1(y_{i}-{\hat{y}}_{i})}{C}\times 100$$

“Categorical cross-entropy” was used as the loss function in hybrid model training and is defined as shown in Equation (6) below. The loss function calculated with the “Categorical cross-entropy” method directly measures the agreement with the real labels for each element (i) [30]:

(6) $$L\left(y,\hat{y}\right)=-\sum_{i=1}^{C}y_{i}\log\left({\hat{y}}_{i}\right)$$

The precision value is calculated using the examples that the model correctly classified as positive (TruePositives—TP) and the examples that it incorrectly classified as positive (FalsePositives—FP), as shown in Equation (7), and the Macro Precision (Equation (8)) value, which expresses the average precision of all classes, is obtained using the number of classes “N”.

(7) $${\mathrm{Precision}}_{i}=\frac{{\mathrm{TP}}_{i}}{{\mathrm{FP}}_{i}+{\mathrm{TP}}_{i}}$$

(8) $$\mathrm{Macro}\;\mathrm{Precision}=\frac{1}{N}+\sum_{i=1}^{N}{\mathrm{Precision}}_{i}$$

In calculating the “Recall” value, in addition to the examples (TruePositives—TP) shown in Equation (9), the examples that the model incorrectly classified as negative are also used, and as in the precision value, the average precision value is obtained as in Equation (10).

(9) $${\mathrm{Recall}}_{i}=\frac{{\mathrm{TP}}_{i}}{{\mathrm{TP}}_{i}+{\mathrm{FN}}_{i}}$$

(10) $$\mathrm{Macro}\;\mathrm{Recall}=\frac{1}{N}+\sum_{i=1}^{N}{\mathrm{Recall}}_{i}$$

The “F1 score” is the metric used to express the balance between two values by taking the harmonic average of Precision and Recall values, as shown in Equation (11).

(11) $$F1=\frac{\mathrm{Precision}\times \mathrm{Recall}}{\mathrm{Precision}+\mathrm{Recall}}$$

### 4.3. Results and Discussion

**Figure 10:**
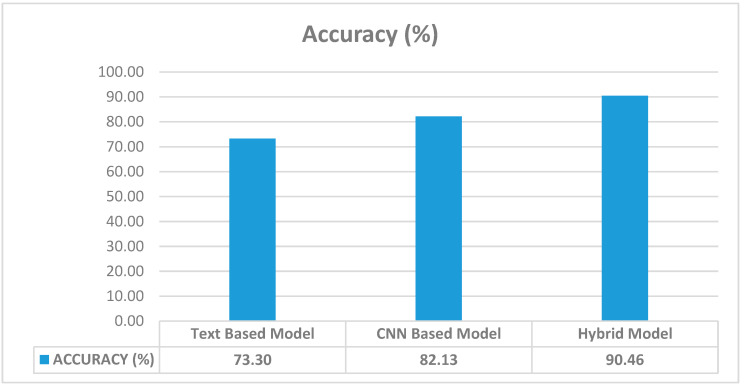
Accuracy graph of text-based model, CNN-based model and hybrid model.

As depicted in Figure 10, the standalone accuracy of the text-based model was 73.30%, while the CNN-based model achieved 82.13%. By combining the features extracted from both models, the proposed two-channel hybrid model attained a notably higher test accuracy of 90.46%. This result demonstrates a significant performance improvement over either model individually, underscoring the effectiveness of the hybrid approach.

The accuracy, precision, recall, and F1 score values of the proposed hybrid model on the test dataset are presented in Table 5. As the results indicate, the model demonstrates balanced and consistent performance across all evaluation metrics. The accuracy of 90.64% reflects the model’s overall effectiveness, while the close values of precision (90.75%) and recall (90.64%) suggest that the model is equally proficient at correctly identifying relevant instances and minimizing false negatives. The F1 score of 90.48% captures this balance, providing a comprehensive summary of the model’s performance.

The MIT Indoor scenes dataset contains a certain imbalance between classes in terms of the number of examples. To reduce the impact of this potential imbalance on test accuracy, weights inversely proportional to the number of examples were assigned to each class during training. This was performed to support the learning of rare scene classes and to balance the impact of overrepresented classes. It was observed that this approach produced positive results in terms of the model achieving high performance early on in the epoch. However, the test accuracy achieved was lower than that of the previous method (89.82%). The accuracy and loss values of the model trained using the weighted loss method are shown in Table 5.

The high and consistent evaluation scores presented in the previous section indicate that hybrid models offer an effective approach for tasks that require rich contextual understanding, such as scene recognition. As highlighted in the literature review (Section 1.2—Related Works), there is a growing emphasis on incorporating local features—particularly object-level information—alongside global representations in indoor scene classification. The proposed hybrid model, aligned with this trend, stands out by achieving superior performance compared to previously reported state-of-the-art methods.

The accuracy values of the models with high success on the MIT-67 dataset published in recent years are shown in Table 6 below [31,32,33,34,35,36,37,38]. Some analyses have been conducted on the classification errors of the hybrid model. One of these analyses reveals the effect of the channel (visual or textual) that influences the final decision in the incorrect example:

When all incorrect classifications in the test data are examined:

- The visual channel is dominant in 72.4% of incorrect classifications.
- In 27.6% of misclassifications, the textual channel is dominant.

This finding shows that most of the model’s incorrect decisions stem from visual representations. In particular, it is estimated that complex backgrounds, visually similar scenes, and low-resolution images mislead the CNN channel.

It is thought that there are different reasons for the misclassification of these classes. The “Related work” section of the study states that high intra-class variety and low inter-class variety make indoor scene classification difficult. Since most scenes have similar architectural and visual structures, the error rates in the visual channel are high. For example, in scenes such as “laboratorywet,” it is estimated that the lack of detailed recognition of sparse objects and technical content by object recognition algorithms, combined with high intra-class variety, has made visual representation difficult for CNN, thereby increasing the error rate. In large-scale areas such as “airport_inside,” many closed-area textures can be encountered at different angles. Similarly, it is thought that large intra-class diversity may have made classification difficult. However, scenes such as “lobby” can be confused with scenes such as ‘waiting_room’ and “living_room,” which are examples of small inter-class diversity. The information demonstrating which channel (visual or textual) was dominant in the incorrect classifications is presented in Table 7.

In order to evaluate the reliability and robustness of the proposed models, all experiments were repeated 10 times with different random seeds. The averages and standard deviations of the accuracy, precision, recall, and F1 score values obtained as a result of these repetitions are presented in Table 8. Accordingly, the average accuracy value of the CNN-based model was calculated as 82.13 ± 0.74%, and that of the text-based model as 73.30 ± 0.81%. The proposed hybrid model outperformed both models, achieving the highest success with an accuracy value of 90.46 ± 0.65. In addition, stable and high results were obtained in all other metrics. These findings show that the hybrid approach is not only effective but also stable in repeated trials.

## 5. Conclusions

In this study, a two-channel hybrid model combining visual (CNN-based) and textual (class description-based) features is proposed to solve the indoor scene recognition problem. The success of the text-based model is enhanced by words obtained from multi-object recognition algorithms, thereby creating more contextual and meaningful class representations. The model’s ability to combine two different types of data significantly improved scene classification performance, surpassing CNN-based single models. The findings indicate that, with the advancement of object recognition algorithms, not only visual but also text-based scene recognition will become more powerful. In particular, it has been observed that text information contributes to model decisions among visually similar classes.

Although the data processing and training processes of the hybrid structure are longer and more costly, the use of multimodal data is an effective approach that is becoming widespread in the field of computer vision. In this context, the proposed method provides a robust and extensible framework for tackling complex tasks that require contextual scene understanding. However, the model’s two-channel structure increases both computation time and memory usage due to the high dimensionality of text features. Additionally, the quality of scene-related textual descriptions and object recognition accuracy are critical factors that directly affect the model’s overall performance. Looking ahead, researching more advanced fusion strategies, such as attention mechanisms, and low-cost structures will further improve the method’s performance and applicability.

## Figures and Tables

**Table 1:** Comparative Overview of the Features of Transformer-Based Architectures and the Proposed Hybrid Model.

Model	Year	Architecture	Modalities	Uses Transformer?	Accuracy (MIT-67, %)	Parameter Count	Remarks
JCVT (Chen Wang et al. [19])	2025	CNN + ViT (LEVTM)	Visual	Yes	98.2	High	Deep hybrid with ViT-based local attention
Li et al. [18] (Scientific Reports)	2025	ResNet50 + SE + Transformer	Visual	Yes	98.4	High	Multiscale fusion with dual-task mechanism
Proposed Model (This Study)	2025	DenseNet121 + Text Classification	Visual + Text	No	90.46	Low	Lightweight, interpretable and modular structure

**Table 2:** Training parameters of CNN-based scene classifier.

Input Width × Height (px)	600 × 600
Train split	80%
Validation split	10%
Test split	10%
Batch size	64
Epoch number	15
Accuracy	82.13%

**Table 3:** Inference values and model accuracies from object recognition models.

Model	Dataset	Number of Classes	Total Number of Objects	Average Number of Objects	Number of Uniq Objects	Model Test Accuracy
Yolov5	Object365	365	320,260	20	262	43.08
Deeplab (Resnet-50)	COCO-Stuff	182	194,190	12	146	44.81
Yolov8	Open Images (V7)	600	251,612	16	350	57.17
Detectron2	LVIS	1200	922,909	59	991	60.53
Extended Model	-	-	1,681,971	107	1206	73.30

**Table 4:** Test performance of the text-based classifier based on the processing applied to word frequencies.

Evaluation on Test Data	Model Without Frequency Sequence Processing	Model with Frequency Sequence Processing
Accuracy (%)	70.1%	73.30%
Loss	1.3246	0.9178

**Table 5:** Evaluation metrics and results of the hybrid model.

Accuracy (%)	90.46
Loss	35.75
Precision (%)	90.85
Recall (%)	90.46
F1 score (%)	90.37
Weighted Accuracy (%)	89.82
Weighted Loss	0.3703

**Table 6:** Comparison of studies conducted in recent years.

Year	Model	MIT Indoor 67 ACC (%)
2020	Semantic-aware [33]	87.10
2020	ARG—Net [38]	88.13
2019	Context modelling with bilstm [10]	88.25
2019	LGN [31]	85.37
2023	CSSRM [36]	88.73
2020	FOSNET [34]	90.30
2024	DGN [35]	90.37
--	Our hybrid model	90.46

**Table 7:** Comparative Overview of Misclassified Samples with Channel Confidence Scores and Dominant Modality in the Hybrid Mode.

Actual Class	Prediction Class	CNN Score	Text Score	Dominant Channel
Lobby	tv_studio	0.0025	0.0018	CNN
bowling	church_inside	0.0024	0.0018	CNN
Office	tv_studio	0.0018	0.0018	CNN
jewelleryshop	mall	0.0018	0.0019	Text
Pantry	corridor	0.0020	0.0020	Text
warehouse	garage	0.0023	0.0018	CNN
operating_room	tv_studio	0.0019	0.0018	CNN

**Table 8:** Performance comparison of the models over 10 repeated runs (mean ± standard deviation).

Model	Accuracy (%)	Precision (%)	Recall (%)	F1 Score (%)
CNN-based Model	82.13 ± 0.74	82.25 ± 0.71	82.13 ± 0.76	82.10 ± 0.73
Text-based Model	73.30 ± 0.81	73.45 ± 0.78	73.30 ± 0.79	73.18 ± 0.82
Hybrid Model	90.46 ± 0.65	90.75 ± 0.62	90.64 ± 0.60	90.48 ± 0.66

## Data Availability

A public dataset was used in this study.
